# The complete chloroplast genome of *Tagetes erecta* (Asteroideae), a wildly cultivated ornamental plant 

**DOI:** 10.1080/23802359.2019.1692704

**Published:** 2019-12-09

**Authors:** Zhao Song, Tian-Qi Li, Zi-Han Liu, Kun Jiang, Xiao-Bin Ou

**Affiliations:** a College of Life Sciences, Zhejiang University, Hangzhou, China;; b College of Life Sciences & Technology, Longdong University, Gansu, China

**Keywords:** *Tagetes erecta*, chloroplast genome, Asteroideae

## Abstract

*Tagetes erecta* (Asteraceae) has been wildly cultivated as ornamental and medicinal plant. Here, we reported the first chloroplast genome sequence of *T. erecta*. The chloroplast genome size is 152,065 bp with GC content of 37.4%, including a large single-copy (LSC) of 83,895 bp, a small single-copy (SSC) of 18,065 bp, and a pair of 25,048 bp IR (inverted repeat) regions. A total of 132 genes were annotated including 87 protein-coding genes, 37 tRNA genes, and 8 rRNA genes. The phylogenetic analysis revealed that *T. erecta* belongs to the subfamily Asteroideae.

*Tagetes erecta* L. (Asteraceae), or Marigold for its common name, is a stout, erect, and branching annual ornamental plant, which grows as a wild and common garden plant world-wide for their gorgeous flowers. Marigold has also been used as medicinal plant, tea, food colorant and in cooking. It has also been reported that Marigold contains diverse secondary metabolites such as flavonoids, sterols, carotenoids and tannins, thus have therapeutic effects on many diseases such as anti-inflammatory (Siriamornpun et al. 2012; Chitrakar et al. 2019). Additionally, flower petals of Marigold are an important source of carotenoids and have been currently taken as the commercial source of lutein (Philip and Berry 1975). Despite its great ornamental and medical importance, there are a few chloroplast markers for breeding of this species. Here, we reported the first chloroplast genome of *T. erecta*, and reconstructed the phylogenetic relationship with other Asteraceae species. The chloroplast genome sequence of *T. erecta* would provide abundant genomic resources for utilization and breeding of this species.

One individual of *T. erecta* was collected from Qingyang, Gansu, China (N35°43′45.3″ E107°41′2.2″). The voucher specimen was deposited in the Herbarium of Zhejiang University (HZU, Accession NO. SZ20190820), Hangzhou, Zhejiang, China. Fresh leaves were dried with silicagel and the total genomic DNA was extracted using a standard CTAB method (Murray and Thompson 1980). Genome sequencing was conducted on HiSeq^TM^2500 (Illumina, San Diego, California, USA) with 150 bp paired-end sequencing. The complete plastome sequence was constructed using GetOrganelle (Jin et al. 2018) and annotated using Geneious Prime 2019.1.1(www.geneious.com) by comparing with the plastome of *Aster indicus* (GenBank Accession No.MG710386), followed by manual inspection. The new annotated plastome sequence was deposited in GenBank (MN462588).

The complete chloroplast genome of *T. erecta* was 152,065 bp length with a GC content of 37.4%. It consists of a pair of IR (inverted repeat) regions of 25,048 bp, separated by a 83,895 bp LSC (large single-copy) and a 18,065 bp SSC (small single-copy) regions. A total of 132 genes were annotated, including 87 protein-coding genes, 37 tRNA genes, and 8 rRNA genes. The IR regions contain 18 duplicate genes (7 protein-coding genes, 7 tRNA genes, and 4 rRNA genes), which including 2 pseudogenes (*ycf1* and *rps19*) in the second IR region.

In order to identify systematic position of *T. erecta,* we conducted a phylogenetic analysis using whole chloroplast genomes of *T. erecta* and the other reported 12 Asteraceae species with *Menyanthes trifoliata* as an outgroup. The sequences were aligned using MAFFT 7.017 (Nakamura et al. 2018). The best-fitting model of nucleotide substitution was GTR + G, as determined by the Akaike Information Criterion (AIC) in jModelTest v. 2.1.7 (Darriba et al. 2012). ML (maximum likelihood) analysis was conducted using RAxML- HPC v. 8.2.8 with 1000 bootstrap replicates on the CIPRES Science Gateway website (Miller et al. 2010). Phylogenetic result strongly supported *T. erecta* belongs to the subfamily Asteroideae (Figure 1), which is consistent with the previous studies based on combined chloroplast genes of Asteraceae (Panero & Funk 2008).

**Figure 1.**
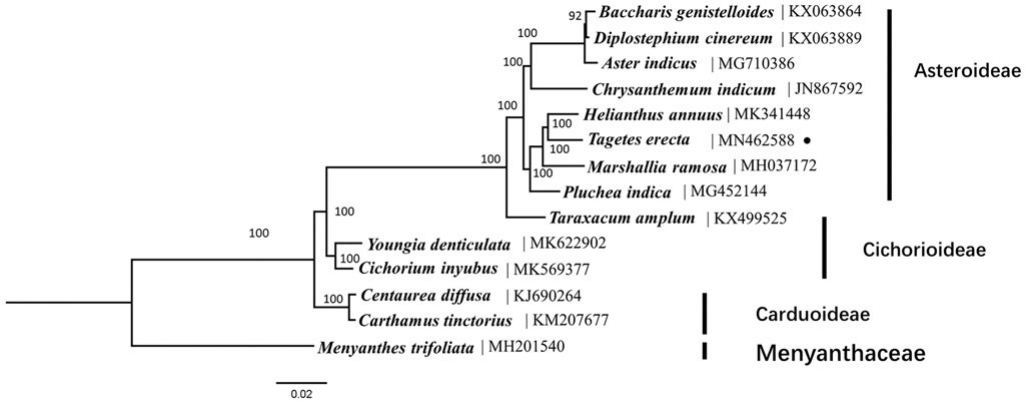
Phylogenetic tree using maximum likelihood (ML) based on plastomes of 13 Asteraceae species and 1 outgroups with 1000 bootstrap replicates. Relative branch lengths are indicated. Numbers near the nodes represent ML bootstrap values.
